# Disease Focused Integrated Care – a New Model of Healthcare Delivery for the Treatment of Skin Cancer

**DOI:** 10.5334/ijic.7009

**Published:** 2023-05-05

**Authors:** Maya Deva, Julie Osborne, Anna McGlynn, Linda Soars, Saleem Loghdey, Kenneth Beath, Peter Gonski, Phil Dwyer, Nicholas Vasudeva, Preeti Joshi, Anand Deva

**Affiliations:** 1 Integrated specialist healthcare education and research foundation, Australia; 2 South Eastern Sydney Local Health District, Australia; 3 New South Wales Agency for Clinical Innovation, Australia; 4 School of Mathematical and Physical Sciences, Macquarie University, Australia

**Keywords:** skin cancer, multilevel, cooperative, integrated care, access

## Abstract

**Introduction:**

As the most common cancer in Australia, skin cancer generates a considerable health burden. This study outlines the establishment of a new model of integrated care for the diagnosis and management of skin cancer.

**Methods:**

A new model of integrated care was established to provide access to all aspects of skin cancer management. General practitioners (GPs) were upskilled through hands-on training and a 6-month skin cancer education program and partnered with specialist Dermatologists and Plastic Surgeons co-located in the same clinic. Data including median wait times between the initial consultation and treatment were prospectively collected and compared patients seen through the integrated pathway to patients referred from their primary GP to specialist Dermatologists and Plastic Surgeons directly (non-integrated pathway). The percentage of patients needing co-consultation with a specialist in the integrated pathway was also measured over time.

**Results:**

A total of 25341 patients were seen from the commencement of the clinic in August 2015 to June 2021. In 2017 and 2018 the median wait time to be treated was 7 days for the integrated model compared to 54 days (2017) and 46 days (2018) for non-integrated care (p < 0.0001). The percentage of GPs requesting specialist co-consultations for assessment of skin cancer fell from 98% in 2015, to 5.6% in 2021. Histopathology shows that 66% of lesions excised by GPs in this model were malignant or pre-malignant.

**Conclusions:**

This study firstly shows a significant reduction in time to treatment in an integrated skin cancer model over traditional models of health. Secondly it demonstrates GP upskilling over time in the integrated program. Integrating GP and specialist medical practitioners in the treatment of skin cancer offers potential for more efficient, accessible, and affordable care. This cooperative, co-located model may provide a template for the integrating the management of other conditions.

## Introduction

Since the introduction of universal healthcare in 1984, Australians have access to some of the best hospitals and standards of care in the world [1,2]. Our life expectancy at 81.5 years is the third highest in the developed world [3,4]. We have well-developed public health programs, good infrastructure, low smoking rates and a population that is generally accepting of health promotion e.g. seat belts and random breath testing [3].

However, ongoing funding of the Australian health sector is projected to hit significant shortfalls [3,5]. The demand and cost for health services will rise, driven by the ageing of the population and increased incidence of chronic disease such as diabetes and obesity [6]. Skin cancer (both melanoma and non-melanoma) has been a longstanding burden on the Australian population and is the leading cause of cancer in the country. The age-standardised incidence of melanoma in Australia has risen from 46.7 to 55.3 cases per 100,000 people from 2001 to 2021 [7]. In 2014, the age-standardised incidence of BCC and SCC was estimated at 271 people diagnosed per 100,000 people [8]. Added to this are the increasing costs of new medical treatments, devices, drugs and interventions and the rising expectations for timely and quality healthcare parallel to rising incomes [9,10,11].

The funding and regulation of the health sector in Australia reflects the complexity of the various organisations involved in healthcare delivery. State and Territory governments are primarily responsible for the management and delivery of major parts of the public health system but much of the funding for this originated in federal taxation revenue. This funding model is currently under negotiation with an ongoing debate about the best way to manage projected shortfalls.

The complexity is further impacted by the interaction between public and private health sector providers. There has been an increased focus on recognizing private benefit and ensuring funding mechanisms limit the incentive for overuse [12]. Several strategies have been suggested along with mechanisms to increase tax revenue specifically to fund healthcare.

These include:

1. Improve efficiency in service delivery and access of patients to care
2. Improving the mix between private and public funding [13]
3. Enhancing preventative strategies to lower disease burden [14]

Integrated care is one model of care that will incorporate these strategies [15,16]. Integrating primary care, specialist medical services and public and private hospital providers in coordinated healthcare delivery has the potential to enable efficiency, cost saving, and improved access to earlier diagnosis and treatment. Integration of private/public sector partners opens avenues to new cross-funding models that may reduce public expenditure. Early models of integrated care delivery have already shown some wide-ranging benefits [17,18,19,20,21] and the integration of health models for cancer treatment have shown additional benefits in the experience and support of patients and families, as well as in treatment outcomes [22].

The NSW government has implemented a policy framework for expanding the use of integrated care in the state. It outlines a vision to integrate care for the entire population from birth to death, optimising the interface between health and social care [23]. We have managed to build from the ground up – a disease-focused model of integrated care in the South Eastern Sydney Local Health District (SESLHD). Our model is guided by the NSW Health principles of creating a patient-centred service that is based around primary care [23]. It provides targeted healthcare services for the treatment of skin cancer and aligns with the values of teamwork and interdisciplinary care. This model has sought to integrate across many levels and has successfully brought engagement of partners across the health sector that are committed to a common primary goal of high-quality, accessible patient-centred care.

This case report aims to outline the establishment of the model and measure its effectiveness using time to definitive treatment of skin cancer and the engagement between primary care physicians and specialists and compare it to the current specialist referral pathway for the treatment of skin cancer. As a proof of concept, we acknowledge that further analysis will be required to establish both medium- and long-term benefits of the model. We acknowledge that the model utilises funding pathways unique to Australia and that scaling of this model to other countries would need to explore alternative sources of partnership and support.

## Methods

### Integrated care model

The integrated skin cancer model was proposed and funded by a one-off innovation grant from NSW Health in 2015. An integrated foundation, Integrated Specialist Healthcare Education and Research Foundation (ISCHERF) was established to help deliver private and public sector partnerships and to obtain funding both for the establishment of the model and for building a purpose-built centre, where the model could be housed. Through a mixture of private sector, not for profit and public funding, a custom designed centre for the delivery of the model was built. This included modular consultation and procedure rooms, a purpose-built pathology laboratory for frozen section analysis and co-location with a day surgery and surgical centre (Ramsay healthcare). Operations commenced at the end of 2015. This model has now scaled to two new sites in NSW – Illawarra Shoalhaven Local Health District & Macquarie University.

Patients were either referred by their primary care physician for assessment or were able to access the integrated model through self-referral. All patients gave consent for their de-identified clinical information to be collected and analysed for the purposes of both clinical audit, health department analysis and research. Patients who were identified through their referral or initial assessment as requiring more urgent action were triaged and prioritised. As demand for the service increased, we ensured that wait times for skin cancer assessment were in line with acceptable practice.

**Figure 1:**
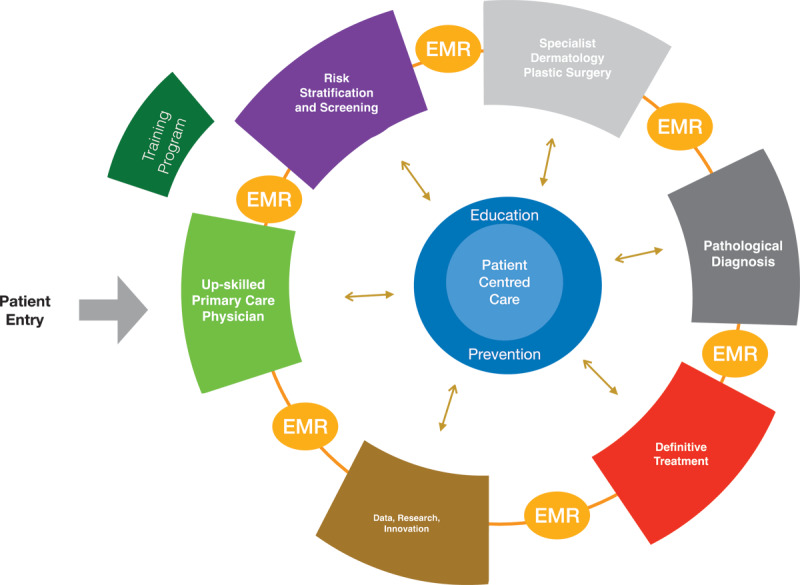
Integrated Skin Cancer Model EMR – electronic medical record.

The skin cancer model is depicted in Figure 1.

In short, the model seeks to place the patient at the centre of the diagnostic and therapeutic circle providing immediate access to primary medical, specialist, diagnostic and therapeutic services at the point of first contact. The models are based on a geographic co-location of these services to ensure that patients are not inconvenienced by having to travel to different locations for access and opportunity for cross consultation on the spot across health disciplines and between GPs and specialists.

### Integration of Care

The integration of care can be classed under four main categories as outlined by Valentijn’s Rainbow Model of Integrated Care: clinical integration, professional integration, organisational integration, and system integration. Professional integration involves utilising inter-professional partnerships to deliver care to a target population, which can be established horizontally through integrating a single-care sector, or vertically through the collaboration between different care sectors [24]. Our skin cancer care model achieves professional integration, where patients in the clinic have access to on-the-spot advice, discussions, and consultations regarding their diagnosis and management from both GPs and specialists in the practice, regardless of who they initially booked to see. This saves patients from re-referrals if other specialist input is needed and promotes collaboration between primary care and specialists.

Improvement in GP skill acquisition was established through a skin cancer education program run by the Primary Health Network (PHN) and SESLHD in partnership with the Integrated Specialist Health Care Education and Research Foundation. Engagement of doctors and health professionals was achieved through the PHN & LHD. This program involved a 6-month training program where GPs attended lectures and workshops. They were also partnered with either specialists or experienced GPs at the clinic, where they were able to develop hands-on skills under supervision. Specialists were available on site to provide advice and a rapid referral pathway. The program was recognised by the PHN who awarded Continuing Personal Development points to participants upon completion. The program clearly articulates educational content and learning objectives. We are currently working with our university to further develop the program and to incorporate an exit assessment to ensure that skill acquisition is measured and validated. This would also allow potential scaling of the program both nationally and internationally.

Recruitment of specialists Dermatologists and Plastic Surgeons proved to be challenging, as there was a reluctance of these specialists to be involved in a cooperative model of care with General Practice. It was fortunate that the model and clinic attracted mid-career specialists who committed to trial the model. In time, the increased workload through the service has provided opportunities to engage and recruit more specialists, especially those that are commencing practice. Feedback from clinicians has been overwhelmingly positive as the model fosters better cooperation and respect between both General Practice and across specialties that traditionally compete for skin cancer work (and revenue). Table 1 outlines the confluence of conditions necessary to support the establishment of the model and their contribution to the model’s success.

**Table 1:** Conditions contributing to the success of the integrated model.


CONDITION	CONTRIBUTION TO SUCCESS

GP education program	Upskilling a primary care workforce to enable quicker and more affordable access

Recruitment of specialists	Providing ongoing specialist input for management of more complex disease. Resource for primary care workforce to continue to upskill

Geographic co-location	Enables real time professional integration. Easier for patients to access clinical, diagnostic and treatment services

Government support	Alignment of policy and funding drivers and support of healthcare administration

Cross funding through procedural and private sector investment	Financial sustainability and means of scaling the model

Risk stratification of patients	Targeting of population at risk and better screening for and prevention of disease


### Statistical Analysis

Prospective data was collected from 2015 to 2021 in the integrated clinic regarding the number of skin cancer consultations by GPs, the number of procedures, and the number of inter-referrals to specialists. For non-integrated patients who had been referred by their primary GP to a Dermatologist or Plastic Surgeon in the practice, the date of the referral and the specialist appointment was recorded prospectively from the practice’s electronic medical record system.

A nested analysis of the waiting times between the initial consultation and treatment by GPs in the integrated clinic and non-integrated pathway was performed from 2017 to 2018. March was selected randomly for the statistical analysis. The time between the initial consultation and treatment for skin cancer was analysed using Kaplan-Meier plots. The comparison between integrated and non-integrated arms was performed using the logrank statistic.

## Results

A total of 25341 patients have been seen through the clinic since its inception in 2015. The efficiency of the integrated model was studied by comparing the percentage of GP consultations that required a specialist co-consultation. In August 2015, 98% of the GP consultations required intra-referrals to specialists. This gradually declined as GPs built up their skills and confidence. From July 2018 to June 2021, the average number of co-consultations that were required was only 5.9%.

In March 2017, there was a total of 61 patients in the non-integrated pathway and 119 in the integrated pathway. For non-integrated patients, the median wait time was 54 days, and the integrated pathway had a median of 7 days, with a significant difference between the two groups (p < 0.0001). In March 2018, 49 patients went through the non-integrated pathway and 125 were integrated. Similar results were seen, with a median wait time of 46 days for non-integrated and 7 days for integrated (p < 0.0001). Integrated and non-integrated groups were compared using logrank statistic. Age and gender were not significantly different between the two groups during those two periods of comparison (See Table 2).

**Table 2:** Summary of Integrated and Non-Integrated patients in March 2017 and 2018.


MARCH 2017	NON-INTEGRATED	INTEGRATED

N	61	119

Age (years)	57.3 (22–83)	59.9 (19–89)

M:F	1.31:1	1.28:1

Median (95% CI)	54 (52,56)	7 (6,7)

Mean (95%CI)	53.1 (50.6,55.7)	5.9 (5.4,6.3)

p value*		<0.0001

March 2018		

N	49	125

Age (years)	57.9 (19–89)	58.6 (21–92)

M:F	1.35:1	1.33:1

Median (95% CI)	46 (42,48)	7 (6,7)

Mean (95%CI)	44.0 (42.0,46.0)	6.7 (6.3,7.2)

p value*		<0.0001


**Figure 2:**
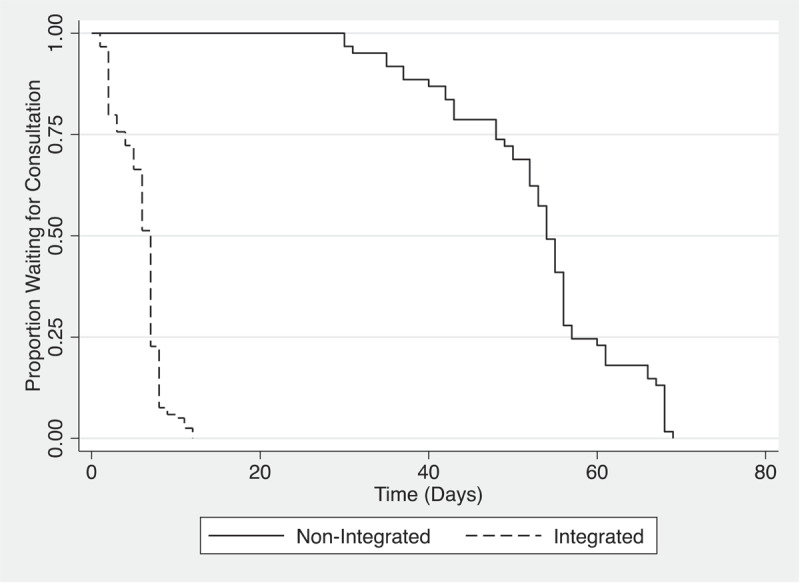
Kaplan-Meier Plot – Waiting time for Consultation.

**Figure 3:**
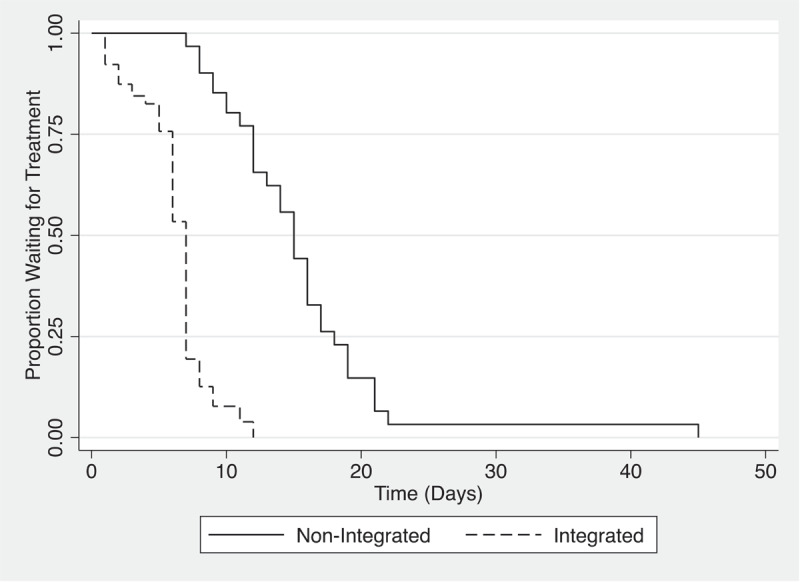
Kaplan-Meier Plot – Waiting time for Treatment.

Kaplan-Meier plots summarising this information are shown in Figures 2 and 3.

**Figure 4:**
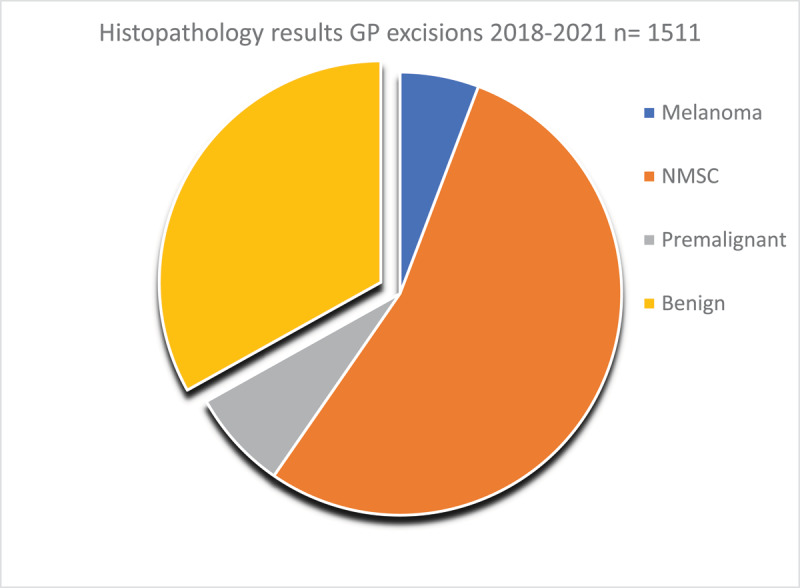
Histopathology from skin lesions excised by GPs in the integrated skin cancer service 2018–2021.

Figure 4 summarises skin cancer pathology treated at the service from 2018 to 2021 arising from procedures. They show that of a total of 1511 procedures performed to excise skin cancers or suspected skin cancers, 66.9% of lesions were either malignant or pre-malignant. Of these, the majority (53.9%) were non-melanoma skin cancers.

## Discussion

This model of disease focused integration has shown some early benefits to patients and healthcare providers. We have shown that time to definitive treatment of skin cancer is significantly faster in an integrated model of care compared with traditional linear care pathways. The benefits of early treatment will need to be further examined to investigate if this translates to a reduction in cost and complexity of treatment and/or reduced risk of recurrent disease.

The integrated model has additionally provided a pathway for GP training in skin cancer care. The requirement for specialist input has decreased steadily over the 7-year period studied, indicating an increase in their confidence and skills. In non-integrated settings, a systematic review showed GPs have significantly worse outcomes for skin cancer excisions than Dermatologists and Plastic Surgeons, with the main reason being a lack of training [25]. Further barriers for GP up skilling identified in a qualitative study include limited time and financial compensation for GPs, and a lack of trust from patients and Dermatologists in GP skills [26]. Improving GP education and implementing structural changes to the healthcare system are ways in which this can be improved, as identified by the study [26]. The integrated model addresses many of these issues through the GP training program and the vertical professional integration. Whilst GPs can upskill in skin cancer diagnosis and management through other educational courses and programs, we speculate that it is the ongoing professional integration with specialist Dermatology and Plastic Surgery using this model that provides for continuous improvement. We have also noted that GPs who work in the integrated centre do take these skills into their general practice setting offering a higher standard of clinical skill in their individual practice locations. In this way, this program may offer a pathway to improve the clinical standards of skin cancer treatment to a wider patient population that those that attend the centre.

The findings of a focused program in integrated care are reflective of the experience overseas where smaller disease centred programs have shown better success in engagement with health workers, administrators, and patients. Other disease programs include diabetes management, haematuria, and bladder disease [6,16]. With our successful engagement of multiple health partners, we envisage similar disease focused integrated care models to be established under this framework of partnership.

The location of the practice in the targets a high-risk group, as the Sutherland Shire has one of the highest rates of melanoma in NSW [27]. Further development of robust risk stratification for skin cancer will allow targeted surveillance and education and has the potential to reduce the incidence of new skin cancers and encourage early diagnosis. We plan to examine reduction in incidence and prevalence of skin cancer in this population to show a measurable benefit of the integrated model.

In addition to the time efficiency for treatment of skin cancer, the integrated model may demonstrate some cost benefits to patients in terms of GP and specialist fees. Whilst economic data is not yet available, the reduction in specialist consultations would also suggest a cost saving to the healthcare system. We are in the process of conducting a detailed health economic analysis to further examine these potential benefits. Furthermore, providing a locally accessible service reduces the cost of travel. This shift may result in reducing the overall cost of skin cancer treatment in the medium to long term.

### Limitations

This study is limited in that the data capturing patient outcomes and their experience for this model is yet to be collected. Shorter wait times, however, may improve patient satisfaction with treatment and access to care. Wait times between integrated and non-integrated patients may have also been influenced by bias if the patients referred between colleagues within the clinic were seen faster as allocation was not randomised. Furthermore, the short sample time for our comparative analysis and potential for referral bias may impact the significance of the findings. These include delayed times to referral and potential geographic difficulty accessing the service. We are currently conducting a more detailed evaluation of the two treatment pathways taking these and other potential confounders into consideration, including the type and stage of skin cancer. We also acknowledge that the centre is located in a metropolitan area and that this model may not be as effective in a rural/regional setting, where GPs may have already developed advanced skills in the management of skin cancer and where specialists are less available. We are currently developing telehealth networks for the management of more complex skin cancer, requiring specialist and multi-disciplinary care with the integrated centre acting as a hub for coordinating both diagnosis and treatment for patients in rural/regional areas.

### Challenges

The scalability of these models has now been proven with similar integrated skin cancer clinics now operational in two other sites in NSW. The engagement of both GPs and specialists delivering care in the same location will naturally create tension between what has been, traditionally, a strict boundary of what treatment is offered by primary care and what requires specialist assessment and treatment. We have managed this by using a collaborative approach to ensure that patients are offered the right treatment by the right clinician at the point of engagement. With increased volume of work, we have seen that all clinicians are busy and that appropriately complex skin cancers e.g., of the head and neck, recurrent tumours or incomplete excisions are fast tracked to specialist treatment. The co-location and vertical integration between GPs and specialists will naturally provide patients with the best option for their care. Discussions between GPs and specialists also reinforces to patients that they are receiving high quality collaborative and appropriate care. We have now used these concepts to develop integrated care models that combine GPs, specialists and allied health targeting chronic disease like obesity and degenerative joint disease and hope to report on their effectiveness in future publications.

## Conclusions

The establishment of an integrated skin cancer service has shown benefits to patient wait times and the clinical integration of skin cancer care. They underlie the urgent need to develop and establish innovative models of healthcare delivery to improve patient access to quality diagnosis and treatment in a wide range of specialties by merging enhanced primary and specialist care in the same location and at the first point of patient engagement. We believe that this model of integrated care ensures that access to universal healthcare is preserved in the face of significant demand and funding pressure.
